# Extracellular volume fraction correlates with myocardial stiffness and allows for differentiation between impaired active relaxation and passive stiffness in heart failure with preserved ejection fraction

**DOI:** 10.1186/1532-429X-18-S1-Q66

**Published:** 2016-01-27

**Authors:** Karl-Philipp Rommel, Maximilian von Roeder, Thomas Stiermaier, Konrad Latuscynski, Christian Oberueck, Stephan Blazek, Marcus Sandri, Christian F Luecke, Matthias Gutberlet, Gerhard Schuler, Philipp Lurz

**Affiliations:** Cardiology, Heartcentre Leipzig, Leipzig, Germany

## Background

Optimal patients characterization in Heart Failure with Preserved Ejection Fraction (HFpEF) is essential to tailor successful treatment strategies.

Cardiac magnetic resonance derived T1-Mapping allows for non-invasive quantification of diffuse myocardial fibrosis as extracellular volume fraction (ECV).

We aimed to elucidate the diagnostic performance of T1-Mapping in HFpEF by examining the relationship between ECV and invasively measured parameters of diastolic function and investigated the potential of ECV to differentiate between different pathomechanisms in HFpEF.

## Methods

We performed T1-Mapping in 21 patients with HFpEF and 11 patients without heart failure symptoms. Pressure-volume-loops were obtained with a conductance catheter during basal conditions and handgrip exercise. Transient preload reduction was used to extrapolate the diastolic stiffness constant.

## Results

Patients with HFpEF showed a higher ECV (p = 0.001), an elevated load-independent passive LV-stiffness-constant β (p < 0.001) and a longer time constant of active LV-relaxation τ (p = 0.04). ECV correlated highly with β (r = 0.75, p <0.001). After multivariate analysis, ECV remained the only independent predictor of β.

Within the HFpEF cohort, patients with ECV > median showed a higher LV-stiffness-constant (p = 0.05) whereas ECV < median identified patients with a prolonged active LV-relaxation (p = 0.01) and a marked hypertensive reaction to exercise due to a pathologic arterial elastance (p = 0.05).

## Conclusions

Diffuse myocardial fibrosis, assessed by CMR derived T1-Mapping, independently predicts invasively measured LV stiffness in HFpEF. In addition, ECV helps to non-invasively distinguish the role of impaired active relaxation and passive stiffness. It also refines characterization of patients, which represents a prerequisite for any successful therapy in the future.

**Figure 1:**
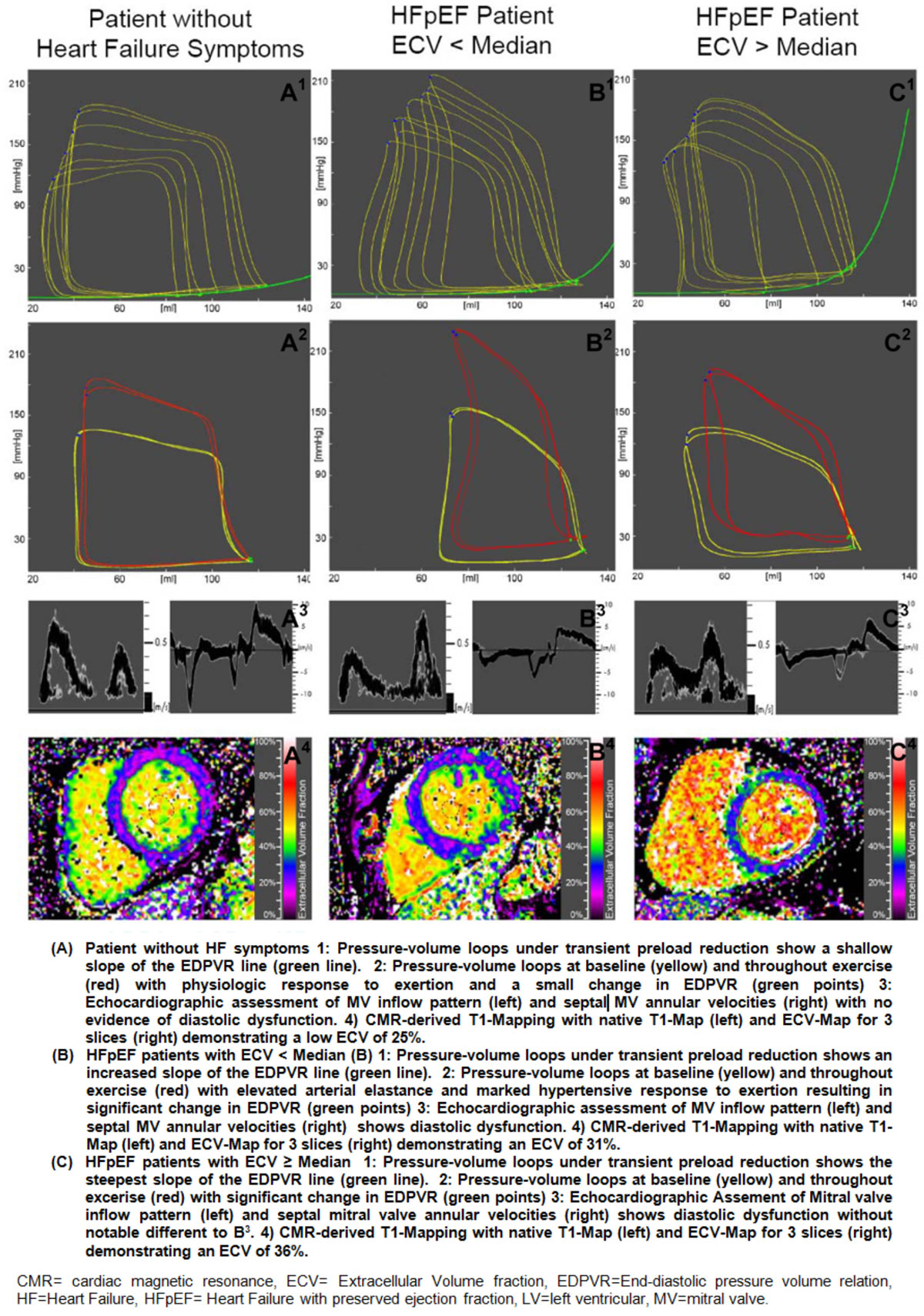
**Results of pressure volume analysis, echocardiography and CMR-imaging according to ECV group and controls**.

